# Associations between lifestyle habits and family members’ health literacy in middle-aged adults with metabolic syndrome: a cross-sectional study in Japan

**DOI:** 10.3389/fpubh.2025.1648414

**Published:** 2025-10-20

**Authors:** Eiko Goto, Hiroko Okada, Hirono Ishikawa

**Affiliations:** ^1^University Hospital Medical Information Network (UMIN) Center, The University of Tokyo Hospital, Tokyo, Japan; ^2^Department of Health Communication, School of Public Health, Graduate School of Medicine, The University of Tokyo, Tokyo, Japan; ^3^Teikyo University Graduate School of Public Health, Tokyo, Japan

**Keywords:** metabolic syndrome, lifestyle factors, family, health literacy, family health education

## Abstract

**Background:**

Health literacy has gained importance as a factor related to health behaviors and the risk of developing metabolic syndrome (MetS). Although the health literacy of family members may affect lifestyle habits among individuals with MetS, few studies have examined these relationships. We examined the associations between lifestyle habits (dietary habits, exercise, drinking, and smoking) among individuals with MetS and their family members’ health literacy.

**Methods:**

We conducted a cross-sectional observational study at a Japanese machine manufacturing company using a self-administered questionnaire. Participants were recruited by the health insurance society based on the criterion of specific health guidance; and participants’ family members were also included in this study. Participants’ lifestyle habits were assessed on the basis of specific health checkup questionnaires. Health literacy was assessed using the Communicative and Critical Health Literacy scale. We used two binary logistic regression models to examine the associations of participants’ lifestyle habits with the health literacy of participants and their family members.

**Results:**

We analyzed data for 125 participants with MetS and 125 family members. After adjusting for variables related to sociodemographic factors and occupation and workplace factors, participants’ exercise habits (moderate exercise, walking/equivalent physical activity) were significantly associated with participants’ health literacy (OR = 4.654, *p* < 0.001 and OR = 2.689, *p* = 0.001, respectively). After adjusting for variables related to sociodemographic factors, occupation and workplace factors, and participants’ health literacy, participants’ dietary habits (time dinner is eaten) and exercise habits (moderate exercise) were independently associated with family members’ health literacy (OR = 2.485, *p* = 0.006 and OR = 2.819, *p* = 0.034, respectively).

**Conclusion:**

The findings suggested that health education for individuals with MetS as well as for their family members may be effective in improving dietary and exercise habits among individuals with MetS themselves. Additional intervention studies are needed to examine which educational model focusing on family is most effective in improving lifestyle habits among individuals with MetS.

## 1 Introduction

Metabolic syndrome (MetS) refers to a cluster of conditions (e.g., abdominal obesity, hypertension, hyperglycemia, elevated triglyceride levels, and reduced high-density lipoprotein cholesterol) that together substantially increase the risk of cardiovascular disease and type 2 diabetes (1). According to the International Diabetes Federation, the global prevalence of MetS among the general adult population is estimated to be between 20 and 25% (2). The World Health Organization also states that non-communicable conditions, including cardiovascular disease, type 2 diabetes and obesity, now account for roughly two-thirds of deaths worldwide (3). Thus, MetS represents an important public health concern.

In recent years, growing attention has been paid to the importance of health literacy in the prevention and management of MetS. Although definitions of health literacy are still evolving, one definition is “the personal skills that enable individuals to obtain, understand, and use information to make decisions and take actions that will impact their health” (4). Previous studies have consistently indicated that individuals with limited health literacy tend to have unhealthy lifestyles, poorer health self-management, and poorer health status (5–8). Thus, health literacy is closely associated with the adoption of lifestyle habits that help to prevent MetS (9).

Individuals with MetS tend to have lower levels of health literacy. Previous studies have suggested that low health literacy is associated with poor disease awareness, insufficient engagement in preventive behaviors such as engaging in physical activity and consuming a healthy diet, and deterioration of physical health status (10, 11). To address this issue, educational interventions (e.g., the distribution of informational pamphlets and verbal counseling) targeting individuals with MetS have been implemented. Previous reports have indicated improvements in clinical indicators such as body weight, waist circumference, and body mass index following lifestyle guidance (12, 13). However, such interventions targeting individuals with MetS are often influenced by individual factors such as receptiveness, motivation, and environmental conditions. Thus, the effectiveness of interventions in sustaining long-term behavioral change and disease management remains limited.

Individuals with MetS are influenced by their family members. Previous research has shown that emotional and practical support from family members—particularly spouses—can contribute to improved dietary and drinking habits, as well as to better clinical outcomes such as improved blood pressure, blood glucose, and lipid levels. (14–16). A previous report also suggested that family members’ knowledge about nutrition and physical activity may promote improvement in the patient’s own lifestyle (17).

Given these findings, family members’ health literacy may influence preventive behaviors and disease management in individuals with MetS. However, no studies to date have assessed health literacy among family members of individuals with MetS. Thus, it is unknown whether lifestyle habits in patients with MetS are associated with the health literacy of their family members. In this study, we aimed to examine the associations between lifestyle habits (dietary habits, exercise, alcohol consumption, and smoking) among individuals with MetS and the health literacy of their family members. The findings are expected to highlight the need for comprehensive support systems that include family involvement, as well as the importance of providing health education targeting family members. This study may contribute to the development of more effective and sustainable support models for the improvement of lifestyles among individuals with MetS.

## 2 Materials and methods

### 2.1 Study design

We conducted a cross-sectional observational study to examine the associations between lifestyle habits (dietary habits, exercise, alcohol consumption, and smoking) among middle-aged Japanese adults with MetS and the health literacy of their family members.

### 2.2 Study participants

Study participants were individuals with MetS who worked at a machine manufacturing company. Since 2008, specific health checkups are conducted by all health insurance societies in Japan for individuals aged 40 years or older with MetS to reduce the risk of cardiovascular disease (18, 19). On the basis of this criterion in the related guidance, our participants were recruited by the health insurance society. Because medical treatments and lifestyle guidance provided by medical practitioners can influence an individual’s lifestyle habits, we excluded individuals who had received medical treatment for blood glucose, blood pressure, and lipids. We finally included 275 individuals with MetS in this study.

### 2.3 Research methods

We conducted a survey using a self-administered questionnaire among the 275 included participants between September 9, 2024 and December 28, 2024. Self-administered questionnaires, instructions and information about participation in this study, and return-mail envelopes were sent to the home of these 275 participants on September 9, 2024 by the health insurance society. The questionnaire was sent again on October 10, 2024 to participants who had not returned a completed questionnaire to their employer. Participants and their family members (members of the participants’ family who were living in the same household and most supportive of participants’ health care) provided written informed consent via the self-administered questionnaires. Through December 28, 2024, a total of 165 out of 275 participants returned a completed questionnaire to the health insurance society (response rate, 59.9%). Among these 165 participants, 134 lived with family members; 125 family members of participants completed the questionnaire (response rate, 93.3%). Thus, 125 participants and 125 family members were included in the analysis.

### 2.4 Measures

#### 2.4.1 Outcomes

We investigated participants’ lifestyle habits (dietary habits, exercise, alcohol consumption, and smoking status) using information provided by the health insurance society based on specific health checkup questionnaires (19). To assess dietary habits, participants were asked, “Do you skip breakfast more than 3 times a week?” and “Do you eat dinner at least 2 h before bedtime at least 3 times a week?” Similarly, to assess exercise habits, participants were asked, “Do you engage in moderate exercise—enough to work up a sweat—for at least 30 min at a time, at least 2 days a week for at least 1 year?” and “Do you perform walking or equivalent physical activity, daily for at least 1 h per day?” Additionally, to measure drinking habits, participants were asked, “How often do you drink alcohol (sake, shochu, beer, other liquor)?” and the amount of alcohol consumed per day, if consumed. Using the Ministry of Health, Labour and Welfare Healthy Japan 21 standards (20), we assessed whether participants’ daily alcohol intake was less than 20 g using their responses. If intake was less than 20 g per day, the participant was defined as drinking an appropriate amount of alcohol. Finally, to measure smoking status, participants were asked, “Do you currently smoke cigarettes?”

#### 2.4.2 Covariates

##### 2.4.2.1 Health literacy

We measured health literacy among participants and their family members using a scale developed and validated in Japan to assess communicative and critical health literacy (21). The scale includes three items on communicative health literacy and two on critical health literacy. Participants were asked whether they could do the following: (i) obtain health-related information from various sources; (ii) extract the required information; (iii) understand and communicate the information obtained; (iv) assess the reliability of the information; (v) make decisions based on the information, specifically in the context of health-related issues. We rated each item on a 5-point Likert scale ranging from 1 (strongly disagree) to 5 (strongly agree). Scores for the five items were summed and divided by 5 to yield a scale score (theoretical range: 1–5). The internal consistency of the scale was adequate (Cronbach’s *α* = 0.86).

##### 2.4.2.2 Personal factors

To investigate participants’ personal factors, we assessed sex (male or female), age (40–49 years, 50–59 years, ≥60 years), education level (junior or senior high school, higher professional school/vocational school/junior college, college or graduate school), marital status (single, married, divorced or widowed), parenting and caregiving responsibilities (parenting responsibilities, caregiving responsibilities, parenting and caregiving responsibilities, no parenting or caregiving responsibilities), relationships with family members (spouse, mother, father, child, other), and household income (<4.0 million JPY, 4.0–8.0 million JPY, >8.0 million JPY) to determine participants’ socioeconomic status.

##### 2.4.2.3 Occupation and workplace factors

To investigate factors related to participants’ occupation and workplace, we assessed their employment status (permanent employee, contracted employee/non-permanent employee), work format (shift work, no shift work), overtime (<1.0 h per day, 1.0–1.9 h per day, 2.0–2.9 h per day, ≥3.0 h per day), and type of work (production line work, clerical position, junior position, managerial position, other).

### 2.5 Statistical analysis

Bivariate association between participants’ and family members’ health literacy was examined using Pearson correlation analysis. We used the independent samples *t*-test to examine univariate associations among participants’ lifestyle habits and participants’ and family members’ health literacy. We then used two binary logistic regression models to examine the associations between participants’ lifestyle habits and the health literacy of participants and their family members. We set each participant’s lifestyle habits as the dependent variable in these logistic regression models. In Model 1, we examined the associations between participants’ lifestyle habits and participants’ health literacy, controlling for seven variables related to sociodemographic factors as well as variables related to occupation and workplace; these variables have been shown in previous studies to be significantly associated with lifestyle factors. In Model 2, we examined the associations between participants’ lifestyle habits and their family members’ health literacy, controlling for the same variables as in Model 1 and participants’ health literacy. We analyzed the data using IBM SPSS version 21.0 (IBM Corp., Armonk, NY, United States).

### 2.6 Ethical considerations

The study was approved by the ethical review committee of the Graduate School of Medicine, The University of Tokyo (examination number 2024174NI).

## 3 Results

Table 1 shows the characteristics and health literacy scores for participants and their family members. A total of 116 (92.8%) participants were men, 62 (49.6%) were in their 40s, 105 (84.0%) were married, and 109 (87.2%) were not shift workers. In 100 participants (80.0%), the family member who completed the survey was the participant’s spouse. The mean participant and family member health literacy scores were 3.58 (standard deviation [SD] 0.71) and 3.52 (SD 0.70). Table 2 shows the bivariate association between participants’ and family members’ health literacy. The health literacy score for family members was positively correlated with the health literacy score of participants (*r* = 0.384, *p* < 0.001). Table 3 shows the univariate associations between participants’ lifestyle habits and participants’ and family members’ health literacy. Among participants, 46 (37.1%) ate dinner at least 2 h before bedtime at least 3 times a week, 38 (30.6%) performed walking or equivalent physical activity each day for at least 1 h daily, and 43 (34.4%) were smokers. We found univariate associations between participants’ health literacy and participants’ exercise habits (moderate exercise and walking or equivalent physical activity). We also found univariate associations between family members’ health literacy and participants’ dietary habits (time dinner is eaten), exercise habits (moderate exercise, walking or equivalent physical activity), and smoking status. Table 4 shows the results of binary logistic regression analysis. After adjusting for seven variables related to sociodemographic factors and occupation and workplace factors, we found that participants’ exercise habits (moderate exercise, walking or equivalent physical activity) were significantly associated with participants’ health literacy (OR = 4.654, *p* < 0.001 and OR = 2.689, *p* = 0.001, respectively) (Model 1). Additionally, we found that participants’ dietary habits (dinner time) and exercise habits (moderate exercise) were independently associated with family members’ health literacy (OR = 2.485, *p* = 0.006 and OR = 2.819, *p* = 0.034, respectively) (Model 2).

**Table 1:** Characteristics of study participants (*n* = 125).

Variables	Total	
	*n*	%
Personal factors		
Sex (*n* = 125)		
Male	116	92.8
Female	9	7.2
Age (years) (*n* = 125)		
40–49	62	49.6
50–59	51	40.8
≥60	12	9.6
Education level (*n* = 125)		
Junior or senior high school	75	60.0
Higher professional school/vocational school/junior college	29	23.2
College or graduate school	21	16.8
Marital status (*n* = 125)		
Single	14	11.2
Married	105	84.0
Divorced or widowed	6	4.8
Parenting and caregiving (*n* = 125)		
Parenting responsibilities	68	54.4
Caregiving responsibilities	7	5.6
Parenting and caregiving responsibilities	3	2.4
No parenting or caregiving responsibilities	47	37.6
Relationship between family members and participants (*n* = 125)		
Spouse	100	80.0
Mother	13	10.4
Father	3	2.4
Child (daughter or son)	7	5.6
Other	2	1.6
Household income (million JPY) (*n* = 125)		
<4.0	11	8.8
4.0–8.0	67	53.6
>8.0	47	37.6
Occupation and workplace factors		
Employment status (*n* = 125)		
Permanent employee	118	94.4
Contracted employee/non-permanent employee	7	5.6
Work format (*n* = 125)		
Shift work	16	12.8
No shift work	109	87.2
Overtime (*n* = 125)		
<1.0 h per day	26	20.8
1.0–1.9 h per day	48	38.4
2.0–2.9 h per day	43	34.4
≥3.0 h per day	8	6.4
Type of work (*n* = 125)		
Production line work	79	63.2
Clerical position	15	12.0
Junior position	13	10.4
Managerial position	14	11.2
Other	4	3.2
Health literacy		
Participants (n = 125)		
Mean (SD)	3.58	0.71
Family members (*n* = 125)		
Mean (SD)	3.52	0.70

SD, standard deviation.

**Table 2:** Correlation between health literacy of participants and their family members (*n* = 125).

	*n*	*r*	*p*
Participants’ health literacy and family members’ health literacy	125	0.384	<0.001

r, Pearson correlation coefficient.

**Table 3:** Univariate associations of participants’ lifestyle habits with health literacy of participants and their family members (*n* = 125).

Variables	Total		Participants			Family members		
			Health literacy score		*p* ^a^	Health literacy score		*p* ^a^
	*n*	%	Mean	SD		Mean	SD	
Dietary habits/breakfast (*n* = 124)								
Does not skip breakfast ≥3 times a week	107	86.3	3.62	0.68	0.116	3.52	0.69	0.857
Skips breakfast at least 3 times a week	17	13.7	3.33	0.87		3.55	0.78	
Dietary habits/dinner time (*n* = 124)								
Does not eat dinner at least 2 h before bedtime at least 3 times a week	78	62.9	3.63	0.76	0.353	3.66	0.70	0.005
Eats dinner at least 2 h before bedtime at least 3 times a week	46	37.1	3.50	0.62		3.30	0.66	
Exercise habits/moderate (*n* = 124)								
Engages in moderate exercise for at least 30 min at a time, at least 2 days a week for at least 1 year	28	22.6	3.96	0.72	0.001	3.90	0.56	0.001
Does not engage in moderate exercise for at least 30 min at a time, at least 2 days a week for at least 1 year	96	77.4	3.47	0.68		3.41	0.71	
Exercise habits/walking or equivalent physical activity (*n* = 124)								
Engages in walking or equivalent physical activity daily for at least 1 h per day	38	30.6	3.88	0.76	0.002	3.72	0.75	0.038
Does not engage in walking or equivalent physical activity daily for at least 1 h per day	86	69.4	3.45	0.66		3.44	0.67	
Alcohol consumption habits (*n* = 125)								
Drinks an appropriate amount of alcohol	108	86.4	3.55	0.73	0.281	3.53	0.69	0.686
Does not drink an appropriate amount of alcohol	17	13.6	3.75	0.55		3.46	0.81	
Smoking status (*n* = 125)								
Non-smoker or former smoker	82	65.6	3.67	0.67	0.053	3.61	0.64	0.044
Smokes every day or occasionally	43	34.4	3.41	0.77		3.35	0.78	

^a^Independent-samples *t*-test. SD, standard deviation.

**Table 4:** Associations between participants’ lifestyle habits and health literacy of participants and their family members (*n* = 125).

Participants’ lifestyle habits	Model 1 (participants’ health literacy)				Model 2 (family members’ health literacy)			
	OR^a^	95% confidence interval lower bound	95% confidence interval upper bound	*p*	OR^b^	95% confidence interval lower bound	95% confidence interval upper bound	*p*
Dietary habits/breakfast Does not skip breakfast ≥3 times a week	1.109	0.634	1.941	0.717	0.723	0.292	1.790	0.483
Dietary habits/dinner time Does not eat dinner at least 2 h before bedtime at least 3 times a week	1.235	0.769	1.982	0.383	2.485	1.299	4.757	0.006
Exercise habits/moderate Engages in moderate exercise for at least 30 min at a time, at least 2 days a week for at least 1 year	4.654	2.157	10.039	<0.001	2.819	1.082	7.344	0.034
Exercise habits/walking or equivalent physical activity Engages in walking or equivalent physical activity daily for at least 1 h per day	2.689	1.482	4.879	0.001	1.487	0.736	3.005	0.269
Alcohol consumption habits Drinks an appropriate amount of alcohol	0.786	0.381	1.623	0.515	1.349	0.574	3.171	0.493
Smoking status Non-smoker or former smoker	1.583	0.966	2.592	0.068	1.403	0.768	2.563	0.271

^a^Odds ratio (OR; healthy lifestyle group compared with unhealthy lifestyle group) were calculated in binary logistic regression analysis adjusted for sex, age, education, household income, employment status, work format, and overtime.

^b^Odds ratios (healthy lifestyle group compared with unhealthy lifestyle group) were calculated in binary logistic regression analysis adjusted for sex, age, education, household income, employment status, work format, overtime, and participants’ health literacy.

## 4 Discussion

In this study, we examined the associations of lifestyle habits among MetS in middle-aged adults and their family members’ health literacy. Compared with the results of specific health checkups conducted for all Japanese citizens aged 40 years and older, the participants in this study had lower rates of skipping breakfast 3 or more times a week, eating dinner at least 2 h before bedtime at least 3 times a week, engaging in moderate exercise, and engaging in walking or equivalent activity each day for at least 1 h daily (22). The mean health literacy scores among participants’ family members were 3.52 (SD 0.70). This score is comparable to the findings of a previous study conducted among 501 pairs of Japanese residents aged 30–79 years and family members with whom participants most often consulted for help with health issues (mean ± SD 3.50 ± 0.77) (23). The health literacy score of family members was positively correlated with participants’ health literacy score. This finding is consistent with those of a previous study that identified a close correlation of family members’ health literacy with participants’ health literacy (23). In the present study, family members’ health literacy was independently associated with participants’ dietary habits (time dinner is eaten) and exercise habits (moderate exercise).

Our study participants who lived in the same household as family members who had better health literacy tended to eat dinner at least 2 h before bedtime, at least 3 times a week, as compared with those whose family members had poorer health literacy. According to a survey conducted by the Statistics Bureau of the Ministry of Internal Affairs and Communications, married Japanese women spend 14.6 times more time managing meals than husbands (24), and it has been reported that many employed women in Japan are responsible for meal management (25). In our study, 80.0% of participants reported that their cohabiting spouse was the person most concerned about their health, and 92.8% of the participants were men. Thus, it is possible that many of our participants were married men living with their wives and that they typically consumed dinners prepared by their spouse. Thus, participants who lived with family members—particularly wives—who had high health literacy may have had dinner served and consumed at appropriate times, regardless of their own level of health literacy. In general, eating dinner close to bedtime is associated with lifestyle-related diseases, including MetS (26). Enhancing the health literacy of family members may reduce the risk of worsening metabolic syndrome and associated cardiovascular diseases among individuals with MetS through optimization of their mealtimes.

Our study participants who lived in the same household as family members with better health literacy tended to engage in moderate physical activity, as compared with those whose family members had poorer health literacy. Previous studies have suggested that family support is a predictive factor of exercise adherence (27) and is associated with persistent or increasing physical activity (28). Previous studies have revealed that individuals benefit from the “distributed nature” of health literacy through shared knowledge, supported skills and practices, supported behaviors, and supported decision-making provided by health literacy mediators belonging to their social networks (23, 29). Thus, participants who lived with family members who had high health literacy may have been more likely to engage in physical activity, as they were more likely to receive support for decision-making and adherence related to exercise from their family members.

This study has several limitations. First, because this was a cross-sectional observational study, we were unable to determine causal relationships among the determinants of participants’ lifestyle habits. Second, participants were recruited from only one company. Additionally, our study population was limited to those over 40 years of age. The generalizability of our findings should be approached with caution based on these sample characteristics. Third, contrary to previous reports measuring functional health literacy, we assessed interactive and critical health literacy. The mean participant health literacy score was 3.58 (SD 0.71), which is lower than that found in a study of Japanese office workers (*N* = 190; mean ± SD 3.72 ± 0.68) (21). The scores obtained in this study were also lower than those in a nationwide online survey of the general Japanese population, which included older adults (*N* = 712; mean ± SD 3.59 ± 0.62) (30). Fewer participants in the present study may have had sufficient health literacy to engage in behaviors to protect their health; thus, participants’ lifestyle may have been more easily influenced by their family members’ health literacy. Fourth, most participants in this study were men. Thus, the findings may have differed if the study population included more female participants. Future research should include women to evaluate potential sex-based differences.

## 5 Conclusion

Despite the above limitations, we explored the associations between lifestyle habits among middle-aged adults with MetS and their family members’ health literacy. In our study, participants’ dietary habits (time dinner was eaten) and exercise habits (moderate exercise) were independently associated with their family members’ health literacy. Our results indicate that health education for individuals with MetS as well as their family members may be effective in improving dietary and exercise habits among individuals with MetS themselves. To address the development of sustainable support models for individuals with MetS, further research is needed to examine the effects of family health education on individuals’ improvement in lifestyles habits.

## Data Availability

The original contributions presented in the study are included in the article/supplementary material, further inquiries can be directed to the corresponding author.

## References

[1] Mottillo S Filion KB Genest J Joseph L Pilote L Poirier P. The metabolic syndrome and cardiovascular risk. A systematic review and meta-analysis. J Am Coll Cardiol. (2010) 56:1113–32. doi: 10.1016/j.jacc.2010.05.034, PMID: 20863953

[2] Alberti KG Zimmet P Shaw J. Metabolic syndrome--a new world-wide definition. A consensus statement from the international diabetes federation. Diabet Med. (2006) 23:469–80. doi: 10.1111/j.1464-5491.2006.01858.x, PMID: 16681555

[3] Riley L Guthold R Cowan M Savin S Bhatti L Armstrong T. The World Health Organization STEPwise approach to noncommunicable disease risk-factor surveillance: methods, challenges, and opportunities. Am J Public Health. (2016) 106:74–8. doi: 10.2105/AJPH.2015.302962, PMID: 26696288 PMC4695948

[4] Nutbeam D Levin-Zamir D Rowlands G. Health literacy in context. Int J Environ Res Public Health. (2018) 15:2657. doi: 10.3390/ijerph15122657, PMID: 30486332 PMC6313523

[5] Papadakos JK Hasan SM Barnsley J Berta W Fazelzad R Papadakos CJ. Health literacy and cancer self-management behaviors: a scoping review. Cancer. (2018) 124:4202–10. doi: 10.1002/cncr.31733, PMID: 30264856

[6] Sørensen K Van den Broucke S Fullam J Doyle G Pelikan J Slonska Z. (HLS-EU) consortium health literacy project European. Health literacy and public health: a systematic review and integration of definitions and models. BMC Public Health. (2012) 12:80. doi: 10.1186/1471-2458-12-80, PMID: 22276600 PMC3292515

[7] Rasu RS Bawa WA Suminski R Snella K Warady B. Health literacy impact on national healthcare utilization and expenditure. Int J Health Policy Manag. (2015) 4:747–55. doi: 10.15171/ijhpm.2015.151, PMID: 26673335 PMC4629700

[8] Kim K Han HR. Potential links between health literacy and cervical cancer screening behaviors: a systematic review. Psychooncology. (2016) 25:122–30. doi: 10.1002/pon.3883, PMID: 26086119

[9] Yokokawa H Fukuda H Yuasa M Sanada H Hisaoka T Naito T. Association between health literacy and metabolic syndrome or healthy lifestyle characteristics among community-dwelling Japanese people. Diabetol Metab Syndr. (2016) 8:30. doi: 10.1186/s13098-016-0142-8, PMID: 27014371 PMC4806481

[10] Peiris C Harding K Porter J Shields N Gilfillan C Taylor N. Understanding the hidden epidemic of metabolic syndrome in people accessing community rehabilitation: a cross-sectional study of physical activity, dietary intake, and health literacy. Disabil Rehabil. (2023) 45:1471–9. doi: 10.1080/09638288.2022.2065540, PMID: 35476590

[11] Peltzer S Hellstern M Genske A Jünger S Woopen C Albus C. Health literacy in persons at risk of and patients with coronary heart disease: a systematic review. Soc Sci Med. (2020) 245:112711. doi: 10.1016/j.socscimed.2019.112711, PMID: 31855729

[12] Shackelford S. Lifestyle intervention in primary care patients with metabolic syndrome. J Clin Lipidol. (2023) 17:e49. doi: 10.1016/j.jacl.2023.05.073

[13] Tejera C Porca C Rodriguez-Carnero G Andújar P Casanueva FF Bellido D. Reducing metabolic syndrome through a group educational intervention program in adults with obesity: IGOBE program. Nutrients. (2022) 14:1066. doi: 10.3390/nu14051066, PMID: 35268040 PMC8912562

[14] Umberson D. Family status and health behaviors: social control as a dimension of social integration. J Health Soc Behav. (1987) 28:306–19. doi: 10.2307/2136848, PMID: 3680922

[15] Lewis MA Rook KS. Social control in personal relationships: impact on health behaviors and psychological distress. Health Psychol. (1999) 18:63–71. doi: 10.1037/0278-6133.18.1.63, PMID: 9925047

[16] Nam HK Chang SJ Kim CB Jeong KS Kim SK Kang DR. The association between social support, metabolic syndrome, and incidence of cardio-cerebrovascular diseases in older adults: the ARIRANG study. Yonsei Med J. (2024) 65:363–70. doi: 10.3349/ymj.2023.0455, PMID: 38804031 PMC11130590

[17] Ho YL Mahirah D Ho CZ Thumboo J. The role of the family in health promotion: a scoping review of models and mechanisms. Health Promot Int. (2022) 37:daac119. doi: 10.1093/heapro/daac119, PMID: 36398941 PMC9673498

[18] Tsushita K Hosler SA Miura K Ito Y Fukuda T Kitamura A. Rationale and descriptive analysis of specific health guidance: the Nationwide lifestyle intervention program targeting metabolic syndrome in Japan. J Atheroscler Thromb. (2018) 25:308–22. doi: 10.5551/jat.42010, PMID: 29238010 PMC5906184

[19] Japanese Ministry of Health, Labour and Welfare. Guidance for smooth implementation of specific health examination and specific health guidance (version 4.1). Available online at: https://www.mhlw.go.jp/stf/seisakunitsuite/bunya/handbook_31132.html (accessed May 13, 2025)

[20] Ministry of Health, Labour and Welfare. Healthy Japan 21 standards. Available online at: https://www.mhlw.go.jp/www1/topics/kenko21_11/b5.html#A52 (accessed May 13, 2025)

[21] Ishikawa H Nomura K Sato M Yano E. Developing a measure of communicative and critical health literacy: a pilot study of Japanese office workers. Health Promot Int. (2008) 23:269–74. doi: 10.1093/heapro/dan017, PMID: 18515303

[22] Survey analysis of the medical insurance group, production department, Federation of Health Insurance Associations. Survey on the questionnaire responses for the specific health examination in fiscal year (2008). Available online at: https://www.kenporen.com/toukei_data/pdf/chosa_r02_11_04-3.pdf (accessed May 13, 2025)

[23] Ishikawa H Kiuchi T. Association of health literacy levels between family members. Front Public Health. (2019) 7:169. doi: 10.3389/fpubh.2019.00169, PMID: 31275918 PMC6593243

[24] Statistics Bureau of Japan. (2017). 2016 survey on time use and leisure activities. Ministry of Internal Affairs and Communications. Available online at: https://www.stat.go.jp/data/shakai/2016/kekka.html (accessed May 13, 2025)

[25] Statistics Bureau of Japan. (2022). 2021 survey on time use and leisure activities. Ministry of Internal Affairs and Communications. Available online at: https://www.stat.go.jp/data/shakai/2021/kekka.html (accessed May 13, 2025)

[26] Yoshida J Eguchi E Nagaoka K Ito T Ogino K. Association of night eating habits with metabolic syndrome and its components: a longitudinal study. BMC Public Health. (2018) 18:1366. doi: 10.1186/s12889-018-6262-3, PMID: 30537972 PMC6288903

[27] Ormel HL van der Schoot GGF Sluiter WJ Jalving M Gietema JA Walenkamp AME. Predictors of adherence to exercise interventions during and after cancer treatment: a systematic review. Psychooncology. (2018) 27:713–24. doi: 10.1002/pon.4612, PMID: 29247584 PMC5887924

[28] Lounassalo I Salin K Kankaanpää A Hirvensalo M Palomäki S Tolvanen A. Distinct trajectories of physical activity and related factors during the life course in the general population: a systematic review. BMC Public Health. (2019) 19:271. doi: 10.1186/s12889-019-6513-y, PMID: 30841921 PMC6404287

[29] Edwards M Wood F Davies M Edwards A. 'Distributed health literacy': longitudinal qualitative analysis of the roles of health literacy mediators and social networks of people living with a long-term health condition. Health Expect. (2015) 18:1180–93. doi: 10.1111/hex.12093, PMID: 23773311 PMC5060848

[30] Ishikawa H Kato M Kiuchi T. Associations of health literacy and information sources with health-risk anxiety and protective behaviors. J Commun Healthc. (2016) 9:33–9. doi: 10.1080/17538068.2015.1133004

